# Genetics and genomic medicine in the Philippines

**DOI:** 10.1002/mgg3.247

**Published:** 2016-09-15

**Authors:** Carmencita D. Padilla, Eva Maria Cutiongco‐de la Paz

**Affiliations:** ^1^ Department of PediatricsCollege of MedicineUniversity of the Philippines ManilaManilaPhilippines; ^2^ Institute of Human GeneticsNational Institutes of HealthUniversity of the Philippines ManilaManilaPhilippines; ^3^ Philippine Genome CenterUniversity of the Philippines SystemQuezon CityPhilippines

## Abstract

Genetics and genomic medicine in the Philippines.

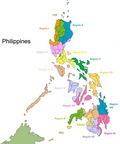

## Demography and Health Indicators

**Figure 1:**
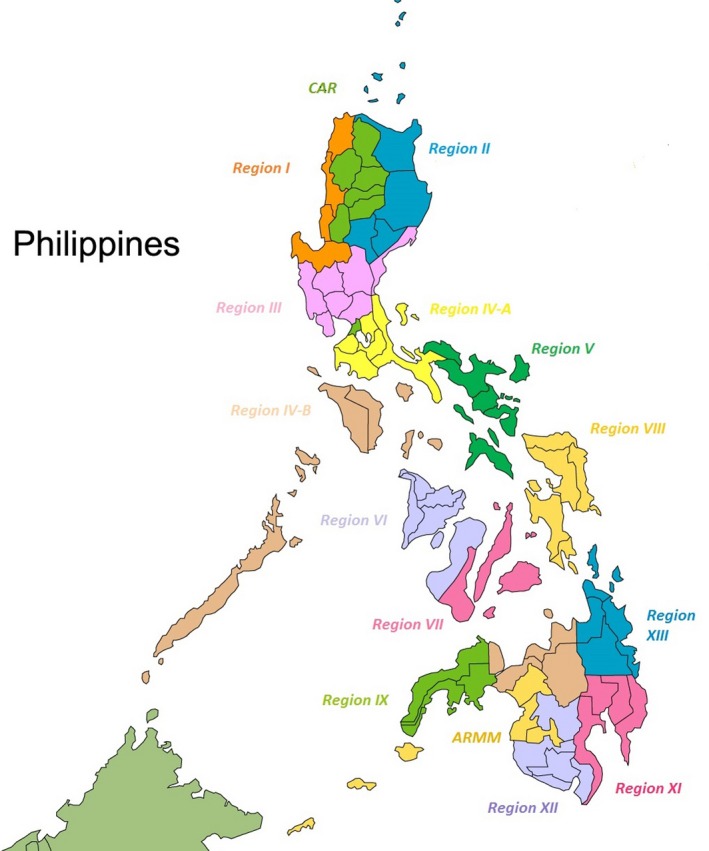
Map of the Philippines.

Located in Southeast Asia, the Philippines is an archipelago covering an area of about 300,000 km^2^ (Fig. 1). The three major island groups—Luzon, Visayas, and Mindanao—are made up of a total of 7107 islands. The country is the 12th most populous country in the world, and 7th in Asia (http://worldpopulationreview.com/continents/asia-population/). In 2016, the population is 104 million, with a population density of 308 persons/km^2^ in 2010 and 342 person/km^2^ in 2016. The majority of the population (54.7%) are in the rural areas (National Statistics Office (NSO) 2015). The annual growth rate is around 1.7%, with a crude birth rate and crude death rate (per 1000 population) of 24.4 and 5.9, respectively (World Health Organization 2015).

Life expectancy is an average of 69 years for both gender (72 years for women, 65 years for men) and has improved since the 1990s. Adult mortality rates (probability of dying between 15–60 years of age per 1000 population) have decreased over the years for both gender (for men: from 272 down to 255; for women: from 154 to 136) (World Health Organization 2015).

The maternal mortality ratio (MMR) remains high but exhibits a decreasing trend. In the early 1990s, the MMR was 152 decreasing to 122 in 1995. In 2015, MMR was 114 per 100,000 live births, with 2700 maternal deaths annually (http://www.who.int/gho/maternal_health/countries/en/). The total fertility rate (TFR) in 2015 was three children per woman (World Health Organization 2015), and in the period of 2007–2012, the TFR declined 1.8% annually (National Demographic and Health Survey 2013).

According to the National Health and Demographic Survey (National Demographic and Health Survey 2013), child mortality in the Philippines is relatively low, compared with other countries in the Southeast Asian region. Under 5 mortality for the years 2009–2013 was 31 deaths per 1000 live births. Infant mortality was 23 deaths per 1000 live births, showing a decreasing trend since 1998. The neonatal mortality rate was 13 deaths per 1000 live births and the postneonatal mortality rate was 10 deaths per 1000 live births. These mortality rates were higher in rural areas than in urban areas (National Demographic and Health Survey 2013). The decreasing trend of child mortality rates may be related to the increase in mothers receiving antenatal care (ANC). In 2013, 95% of women were reported to have received ANC from a skilled provider: 55% was provided by a midwife, 39% by a doctor, and 2% by a nurse. Less than 1% of women received ANC from a traditional birth attendant (TBA), whereas 4% did not receive any antenatal care. These results indicate that there has been an increase in the proportion of births attended by a skilled provider (from 9% in 2008 to 95% in 2013) and a decline in the proportion of women who obtained TBA services (from 5% in 2008 to <1% in 2013). Additionally, there has been an increase in births in health facilities, from 44% in the 2008 NDHS to 61% in 2013. Of these health facility births, 43% were provided by the public sector. A corresponding decline was seen in home deliveries, from 56% in 2008 to 38% in 2013 (National Demographic and Health Survey 2013).

## Health Expenditure and Financing

The country's total health expenditure (THE) went up by 11.7%, from Php 471.1 billion in 2012 to Php 526.3 billion in 2013. This could be attributed to the large amount expended for health care by private sources, the majority of which (82.6%) were disbursed by households (private out‐of‐pocket) for their health needs in 2013 (Philippine National Health Accounts 2013). Private sources remained the biggest contributor to THE, accounting for 68.2% of the total outlay for health for that year. Government expenditures came in a far second with 18.9%, followed by social insurance with 11.5% and the rest of the world with 1.4% (http://www.nscb.gov.ph/stats/pnha/dataCharts.asp).

## The Burden of Birth Defects and Genetic Diseases in the Country

Infectious diseases are still the most common causes of infant mortality and morbidity in the Philippines. Although birth defects also have been among the top causes of infant mortality since the 1960s, limited attention has been given to birth defects and other genetic conditions (http://www.doh.gov.ph/mortality). The situation has been aggravated by the small number of geneticists and genetic counselors who can provide genetic diagnosis, management, and counseling services to patients. The delivery of medical genetic services thus remains a challenge in both private and public sectors. The March of Dimes reported the estimated prevalence of congenital disorders was 52.9 per 1000 live births in 2006 with details presented in Table 1 (Christianson et al. 2006).

**Table 1:** Prevalence of congenital disorders by cause

Cause	Estimated prevalence (per 1000 live births)
Dominant single‐gene disorders	7
Recessive single gene disorders	2.3
X‐linked recessive single‐gene disorders	1.3
Chromosomal disorders	4.2
Malformations	63.9

Source: Christianson A, Howson CP, Modell B (2006), March of Dimes (MoD) Global Report on Births Defects, 2006: The Hidden Toll of Dying and Disabled Children.

There have been three major attempts to set up a birth defects registry in the country. In 1999, the Institute of Human Genetics, National Institutes of Health (IHG‐NIH), University of the Philippines (UP) Manila, in partnership with the Department of Health (DOH), conducted a pilot project of a birth defects registry. This project involved examination of 191,576 hospital‐born newborns in 79 hospitals which revealed a total of 1240 birth defect cases. The data were given to the DOH, but unfortunately the birth defects registration was not integrated into their system (Padilla et al. 2003). In 2008, a birth defects surveillance project was conducted in 82 facilities under the Global Network for Maternal and Infant Health supported by the March of Dimes, USA. In addition to birth defects registration, prevention campaigns were conducted by the Volunteer Youth Leaders for Health, whose advocacy is birth defect prevention. The major limitation of this project was the underreporting of birth defect cases in the participating hospitals and communities (Padilla et al. 2011a). The third birth defects registry is a 10‐year review at the Philippine General Hospital (PGH), the biggest government hospital (Padilla et al. 2011b). The limitation of this project is the bias toward the admission of high‐risk cases being a referral hospital.

## Availability of Genetic Services

Many genetic tests and services are available from and delivered to the whole country by the IHG‐NIH and limited government and private institutions. The IHG‐NIH provides clinical genetics services and the following genetic testing services: cytogenetics, molecular genetics, biochemical genetics, and newborn screening. The Institute is also involved in research and various advocacies (Padilla 2008; Padilla et al. 2012; Padilla and Cutiongco‐de la Paz 2013).

### Clinical genetics

The Clinical Genetics Unit provides comprehensive clinical services to families and individuals with, or at risk for, an inherited disease. It offers genetic diagnoses, management, and genetic counseling to families. Clinical genetics services are rendered at the Philippine General Hospital (PGH) Department of Pediatrics. Services are provided by geneticists, genetics fellows‐in‐training, pediatric residents rotating at the Clinical Genetics Unit, and nurses. An average of 1500 cases is served each year. The activities of the unit include: (1) clinical genetics outpatient service (6 half‐day clinics per month) receiving intra‐ and interhospital and referrals from private physicians; (2) genetic metabolic clinics (2 half‐day clinics per month) to take care of patients with inborn errors of metabolism, including enzyme replacement therapy for lysosomal storage disorders (LSDs) such as Gaucher disease, Pompe disease, and Mucopolysaccharidosis; (3) special genetic metabolic clinics (every 6 months) where patients who need multidisciplinary care are seen by a multidisciplinary team such as for Osteogenesis Imperfecta and Mucopolysaccharidosis (Padilla 2008; Padilla et al. 2012; Padilla and Cutiongco‐de la Paz 2013); and (4) support group meetings. The top diagnoses made in the outpatient clinics and inpatient referrals are Down syndrome, metabolic disorders [maple syrup urine disease (MSUD) is most common], developmental delay, and multiple congenital anomalies. Clinical genetic services are also available in the private clinics of the clinical geneticists in Manila, Cebu, and Davao.

### Cytogenetics

The Cytogenetics Unit of IHG‐NIH performs routine karyotyping of peripheral blood cells, cord blood, solid tissues, and bone marrow for patients with multiple birth defects, mental retardation, abnormal sexual development, for couples with infertility or multiple miscarriages, and for patients with malignancies and hematologic disorders. It also offers high‐resolution banding and fluorescence in situ hybridization (FISH) analysis for testing other chromosomal disorders and cancers (Padilla and Cutiongco‐de la Paz 2013). Chromosomal microarray analysis for selected clinical cases is sent to overseas laboratories. Cytogenetic services are also available at the Philippine Nuclear Research Institute, the Research and Biotechnology Division of St. Luke's Medical Center, and National Kidney and Transplantation Institute.

Of 14,687 peripheral blood samples received by the Cytogenetics Unit of the IHG‐NIH from 1991 through 2015, 5117 (34.8%) revealed abnormal results: 482 cases of sex chromosome numerical abnormality, 48 cases of sex chromosome structural abnormality, 4051 cases of autosomal numerical abnormality, and 536 cases of autosomal structural abnormality (Table 2).

**Table 2:** Distribution of chromosomal abnormalities seen at the Cytogenetics Laboratory, IHG‐NIH (1991–2015)

Numerical abnormalities of the sex chromosomes		482
XXX and variants	19	
XXY and variants	45	
XYY	4	
Turner syndrome		
45,X	153	
Turner syndrome variants	261	
Structural abnormalities of the X and Y chromosome		48
X;Autosome translocations	8	
Balanced translocations (6)		
Unbalanced translocations (2)		
Isochromosome Xp	1	
Isodicentric Xp chromosomes	3	
Isodicentric Xq chromosomes	2	
Structural abnormalities of the Y chromosome	31	
46,XX/46,XY	3	
Numerical abnormalities of the autosomes chromosomes		4051
Autosomal trisomies	4036	
Trisomy 21 all types (3476)		
Trisomy 18 all types (455)		
Trisomy 13 all types (92)		
Trisomy 8 (2)		
Trisomy 9 all types (5)		
Trisomy 11 (1)		
Trisomy 22 all types (5)		
Monosomy 21	5	
Triploidy	1	
Double anueploidy	9	
48,XXX,+18 (4)		
48,XXY,+21 (4)		
48,XYY,+21 (1)		
Structural abnormalities (addition, deletion, duplication, insertion, inversions, isochromosomes, ring chromosomes, translocations, fragile sites and marker chromosomes		536
Total		5117

### Molecular genetics

The Molecular Genetics Unit of IHG‐NIH conducts interdisciplinary research in collaboration with both local and international academic clinicians and scientists with specialist groups studying the genetics of monogenic and complex genetic conditions in the Filipino population. Using molecular genetics techniques, new diagnostic tools are applied for the conditions seen in the clinics to understand how these genes cause disease (Padilla 2008; Padilla et al. 2012; Padilla and Cutiongco‐de la Paz 2013). IHG‐NIH has a DNA sequencing facility, a microarray facility, and a bio‐bank facility. For tests that are not available in the country, it facilitates the DNA extraction and transport of samples to overseas laboratories. Pregenetic testing and postgenetic testing counseling are provided by geneticists and genetic counselors. Some private hospitals are also able to offer molecular‐based testing for cancer.

The Molecular Genetics Laboratory of IHG‐NIH has focused its services on molecular‐based testing for the more common genetic conditions among Filipinos such as cancer, cardiovascular disease and X‐linked Dystonia Parkinsonism (XDP) as well as those genetic disorders which are part of the newborn screening program, such as MSUD, Phenylketonuria (PKU), Methylmalonic Acidemia (MMA), Congenital Adrenal Hyperplasia (CAH), Glucose‐6‐Phosphate Dehydrogenase (G6PD) Deficiency, alpha and beta‐thalassemia. Many of the molecular‐based projects are closely linked to the Philippine Genome Center (PGC), University of the Philippines.

The studies on MSUD have identified the most common gene deletion among Filipinos—a 4.6 kb deletion of the dihydrolipoamide branched chain transacylase E2 gene *(DBT)* (OMIM 248610, Genbank NM_001918). Mutation testing has been beneficial to families for early diagnosis and management of affected members to prevent permanent neurological impairment (Silao et al. 2004, 2008). Filipino PKU patients have been found to have previously identified p.I65T, p.R413P, p.Ex6‐96A>G, and p.R243Q mutations in the phenylalanine hydroxylase gene (*PAH*) (OMIM 612349, Genbank K03020). This has significantly aided in the medical management and genetic counseling of patients and their families (Silao et al. 2009a). The molecular genotyping of Filipino patients with MMA showed compound heterozygosity for c.1595G>A, c.2011A>G, c.322C>T, c.982C>T, and c.1280G>A, allowing directed mutation analyses for the local population (Silao et al. 2009b). Another common genetic disorder, CAH, was found to have an estimated crude incidence higher than that reported in most populations. Studies that used a method of combined differential PCR and amplification created restriction site approach, and direct probing for the presence of known mutations in the cytochrome P450 family 21 subfamily A member 2 gene (*CYP21A2*) (OMIM 613815, Genbank NM_000500) and cytochrome P450 family 21 subfamily A member 1 pseudogene (*CYP21A1P*) (Genbank KC493621) of Filipino CAH patients showed a majority of cases with a premature splicing error mutation. Determination of frequent alleles facilitates rapid screening for mutations in the 21‐OH gene and leads to a definitive diagnosis and possible prenatal intervention (Cutiongco‐de la Paz et al. 2009). Molecular characterization of the glucosylceramidase beta gene (*GBA*) (OMIM 606463) among Filipino patients with Gaucher disease revealed compound heterozygotes with the following genotypes p.L444/p.P319A and p.L444P/p.G202R. Genotype–phenotype correlation showed that these genotypes are compatible with the severe neuronopathic type of Gaucher disease (Chiong and Silao 2011). Galactose‐1‐phosphate uridylyltransferase gene (*GALT*) (OMIM 606999) testing of unrelated Filipino patients with Classic Galactosemia showed missense mutations; the p.V168L and p.A345D genotypes have already been reported previously, but the p.L116P and p.M178R mutations appear to be novel mutations with the p.V168L being the most frequent mutation. This study suggests that galactosemia is a heterogeneous disorder at the molecular level and that *GALT* mutations are ethnic specific (Estrada et al. 2013). The first mutational analysis of cystathionine *β*‐synthase gene (*CBS*) (OMIM 613381) deficiency was done in a Filipino patient with classic homocystinuria. The patient was found to be compound heterozygous for two novel mutations g.13995G>A [c.982G>A; p.D328K] and g.15860‐15868dupGCAGGAGCT [c.1083‐1091dupGCAGGAGCT; p. Q362‐L364dupQEL] (Silao et al. 2015a). Using multiplex polymerase chain reaction (PCR) with multiple tandem forward primers and a common reverse primer (MPTP), G6PD variants were detected with the G6PD Viangchan being the most common, followed by the G6PD Union, G6PD Vanua Lava, G6PD Chatham, and G6PD Canton (Padilla et al. 2011c).

A molecular epidemiology study that was designed to look for the association of environmental factors and specific genetic polymorphisms with cancer susceptibility was also undertaken. The study's objective was to identify genetic polymorphisms that could be potential biomarkers for breast, colorectal, and oral cavity cancer in the Filipino population. For oral cavity cancers in particular, the glutathione S‐transferase pi 1 gene (*GSTP1)* (OMIM 134660, Genbank NM_000852) c.313A>G homozygous genotype was found to be associated with cancer risk when adjusted for epidemiologic risk factors identified (Cutiongco‐de la Paz et al. 2013). In the same study, epidemiological risk factors that were identified to be significantly related to the cancers include: cigarette smoking for lung and oral cavity cancer; passive smoking, tobacco chewing, and inverted cigarette smoking for oral cavity cancer; family history of cancer for lung and breast cancer; and increasing age at first pregnancy for breast cancer. These findings are consistent with data from other countries (Ngelangel et al. 2009). High breast cancer incidence rates have been reported in Southeast Asia with the Philippines having one of the highest rates. A case–control study on Filipinos revealed that *BRCA* mutations had a prevalence of 5.1%, with 4.1% of the mutations on the *BRCA2* DNA repair‐associated gene (*BRCA2*) (OMIM 600185, Genbank U43746). This is attributed to the presence of two common founder mutations in *BRCA2* among Filipino women with breast cancer (De Leon Matsuda et al. 2002). Another study on cancer, specifically of the colon and rectum, revealed that the incidence of *ras* mutations among Filipinos is lower than other populations (Carillo et al. 2009).

Studies on determining the prevalence of hemoglobinopathies in the Philippines have also been done using high‐performance liquid chromatography (HPLC). The majority of the patients have beta thalassemia followed by alpha‐thalassemia. Hemoglobin E was found in 1% of the population tested whereas 2% of the patients have beta thalassemia with HbE interaction. These results confirm the presence and prevalence of these hemoglobinopathies in the Filipino population (Silao et al. 2015a, b).

Studies to identify genes among Filipinos that put the population at risk for cardiovascular disease have also been done. One of these studies involves the association of mutations on the low‐density lipoprotein‐receptor gene (*LDLR*) (OMIM 606945, Genbank DQ379956) that is related to Familial Hypercholesterolemia (FH). There were six novel mutations identified, and it was found that there is a significant association between mutations and number of clinical variables, including family history of dyslipidemia, FH score, and LDL‐cholesterol level (Punzalan et al. 2005). Another study aimed to associate cholesterol ester transfer protein (TaqIB) polymorphism with HDL‐C levels among Filipinos. The frequencies of polymorphisms were 40% for B1B1, 50% for B1B2, and 10% for B2B2. Additionally, B1B1 is found to be associated with low HDL‐C levels. With the identification of these polymorphisms, causes of low HDL‐C other than the traditional causes among Filipinos have been established (Sy et al. 2007).

The Institute was also involved in a landmark paper conducted by more than 90 scientists from 11 Asian countries who joined the HUGO Pan Asian SNP Consortium. The consortium embarked on a human genetic mapping study of 73 Southeast Asian (SEA) and East Asian (EA) population. Findings showed that genetic ancestry is highly correlated with linguistic affiliations and geography. The study suggested that there was a single primary wave of entry of humans into the Asian continent. (HUGO Pan‐Asian SNP Consortium 2009). The Philippines continues to be a part of the HUGO Consortium, now known as the HUGO Pan‐Asian Population Genomics Initiative or PAPGI, a collaborative effort to systematically collect, analyze, and understand the genetic diversity of the Asian populations. The data generated will be made publicly available to the worldwide scientific community for further studies on human evolution and medical applications (http://www.hugo-international.org).

### Biochemical genetics

The Biochemical Genetics Unit of IHG‐NIH offers expert diagnostic testing and provides physician‐assisted consultative services to clinicians in order to provide accurate diagnosis and appropriate management for patients with inherited metabolic disorders. The array of biochemical tests include urine metabolic screen by high‐voltage electrophoresis, quantitative amino acid analysis by ultrahigh‐performance liquid chromatography, urine organic acid analysis by gas chromatography‐mass spectrometry, plasma acyl carnitine analysis by liquid chromatography‐tandem mass spectrometry and tests for LSDs, mitochondrial disorders, and peroxisomal disorders that are sent out overseas. It serves as a reference laboratory for the national expanded newborn screening program for confirmation of amino acid, organic acid, and fatty acid oxidation disorders. Table 3 shows the number of patients with metabolic disorders diagnosed at the Biochemical Genetics Laboratory of IHG‐NIH.

**Table 3:** Metabolic registry, biochemical genetics laboratory, IHG‐NIH from 1999 to 2016

Disorder		No. Cases
Amino acid disorders		215
Maple syrup urine disease	155	
Hyperphenylalaninemia	57	
*Phenylketonuria (25)*		
*Mild hyperphenylalaninemia (24)*		
*6 Pyruvoyltetrahydropterin synthase deficiency (8)*		
Tyrosinemia I	1	
Non ketotic hyperglycinemia	1	
Homocystinuria	1	
Lysosomal storage disorders		80
Mucopolysaccharidosis (MPS)	57	
*MPS Type I (2)*		
*MPS Type II (41)*		
*MPS Type III‐B (1)*		
*MPS Type IV (7)*		
*MPS Type VI (2)*		
*Unclassified (4)*		
Gaucher disease	11	
Pompe	4	
Fabry disease	3	
Mucolipidosis	1	
LSD – Multiple sulfatase deficiency	1	
Ceroid lipofuscinosis neuronal 2	1	
Tay Sach's disease	1	
Niemann pick	1	
Galactosemia		86
Classical	18	
Non‐classical	*68*	
Organic aciduria		25
Methyl malonic aciduria (MMA)	17	
Glutaric aciduria type I	4	
Multiple carboxylase deficiency (MCD)	2	
L‐2‐Hydroxyglutaric aciduria	1	
3‐Methylcrotonyl‐Coa carboxylase deficiency (3MCC)	1	
Urea cycle defects		7
Argininosuccinate lyase deficiency syndrome (ASALD)	1	
Ornithine transcarbamylase (OTC) deficiency	1	
Carbamoyl phosphate synthase (CPS) deficiency	5	
Fatty acid oxidation disorders		3
Very long chain acyl CoA dehydrogenase deficiency (VLCADD)	*2*	
Medium chain acyl CoA dehydrogenase deficiency (MCAD)	1	
Mitochondrial respiratory chain disorders		3
MELAS	2	
Respiratory chain complex/deficiency	1	
Others		36
Adrenoleukodystrophy (ALD)	21	
Homozygous cystinuria	3	
Heterozygous cystinuria	9	
Lowe syndrome	1	
Lesh nyhan disease	1	
Menkes	1	
Total		455

### Newborn screening

The most successful population‐based genetic screening program in the Philippines is newborn bloodspot screening (NBS). NBS was introduced in the Philippines in 1996 by obstetricians and pediatricians from 24 private and government hospitals. NBS was integrated into the public health delivery system with the enactment of Republic Act 9288 or Newborn Screening Act of 2004. This Act institutionalized the “national comprehensive NBS System (NCNBSS),” which ensures that: (1) every baby born in the Philippines is offered NBS; (2) the establishment and integration of a sustainable NBS System within the public health delivery system; (3) all health practitioners are aware of the benefits of NBS and of their responsibilities in offering it; and (4) all parents are aware of NBS and their responsibility in protecting their child from any of the disorders. The very first Newborn Screening Center in the country was established at the IHG‐NIH (NSC‐NIH). It administers both laboratory testing and follow‐up services. To date, there are five (5) NSCs that are strategically located throughout the country: NSC‐NIH, NSC‐Central Luzon, NSC‐Visayas, NSC‐Southern Luzon, and NSC‐Mindanao. Another four NSCs will be set up in the country.

From 1996 to December 2015, a total of 7,709,243 newborns were screened, with more than 140,000 patients saved. As of December 2015, 77% of the estimated 1.8M total live births in the country were screened, 12% more than the previous year. More than 6000 health facilities (birthing centers, lying‐in clinics, rural health units, infirmaries, secondary/secondary/tertiary hospitals) offer NBS. These facilities educate parents about NBS during prenatal visits, perform blood sample collection for NBS, organize transport of samples to NSCs, recall patients with positive screening results, and assist in the referral and management of patients. Expanded Newborn Screening (ENBS) offering an additional 20 disorders was begun in 2014 including additional testing for hemoglobinopathies, amino acid disorders, organic acid disorders, endocrine disorders, biotinidase deficiency, and cystic fibrosis (www.newbornscreening.ph).

Table 4 shows the number of confirmed cases of various metabolic and endocrine conditions as well as hemoglobinopathies since the start of the expanded newborn screening program.

**Table 4:** Prevalence of disorders among Filipino newborns (1996–2015)

Disorder	No. of confirmed cases	Prevalence
Congenital hypothyroidism^a^	2793	1: 2680
Congenital adrenal hyperplasia^a^	481	1: 15,560
Galactosemia^a^		
Classical (GALT)	21	1:356,391
Nonclassical	71	1:105,411
Duarte variant	89	1:84,092
Phenylketonuria^a^		
Classical	13	1:575,709
Mild PKU	14	1:534,587
BH4 Def	7	1:1,069,173
Hyperphenylalaninemia	30	1:249,474
Maple syrup urine disease^b^	47	1:82,354
G6PD deficiency^a^	136,524	1:54
Medium chain‐acyl‐CoA dehydrogenase deficiency (MCAD)^c^	1	1:50,262
Multiple carboxylase deficiency (MCD)^c^	1	1:50,262
Methylmalonic acidemia (MMA)^c^	1	1:50,262
Very long chain‐acyl‐CoA dehydrogenase deficiency (VCAD)^c^	1	1:50,262
HbH disease alpha thalassemia^c^	42	1:1169
Hemoglobin E disease^c^	1	1:50,262

a Basic Panel of 6 tests with n 7,709,243.

b MSUD was introduced in 2012 with n 2,870,648.

c ENBS started 2014 with n 50,262.

Short‐term follow‐up of the patients is handled by the NSCs and once diagnosis is confirmed, they are endorsed to one of the strategically located regional Newborn Screening Continuity Clinics providing long‐term follow‐up management.

### Prenatal diagnosis

Prenatal diagnosis is practiced in a very limited way. The most extensively used prenatal diagnostic procedure is prenatal ultrasonography, which utilizes two‐dimensional ultrasound for congenital anomaly screening during the second trimester of pregnancy. This is complemented by fetal echocardiography in cases of congenital heart defects detected after a congenital anomaly scan. First trimester ultrasound screening has been introduced in some urban medical centers. More advanced techniques of three‐ and four‐dimensional ultrasonography and color‐Doppler ultrasound have recently gained popularity. Maternal serum screening, using single or multiple markers as noninvasive forms of prenatal diagnosis, has not been customarily offered in the country (Cutiongco‐de la Paz 2006). Amniocentesis is offered only for anticipatory guidance for obstetricians and pediatricians for a problematic pregnancy or preparation for the birth of a child with birth defects. Chorionic villi sampling has not been normally offered in any institution. Termination of pregnancy is not allowed by law in the country (Cutiongco‐de la Paz 2006).

## Access to Genetic Services in the Philippines

In general, 48.4% of health expenditure is out‐of‐pocket. Among the genetic tests, NBS is the only one covered by the national health insurance. All other expenses related to treatment upon diagnosis are out‐of‐pocket expenses for the family. The following are barriers to accessing genetic services in the Philippines: (1) financial, as most families cannot afford out‐of‐pocket expenses for the expensive genetic testing and treatment; (2) geographical, being an archipelago of 7107 islands; (3) lack of awareness among different stakeholders, that is, health professionals and parents; (4) compromised access to genetic services at the regional and provincial level; and (5) lack of geneticists and genetic counselors.

## The Future of Medical Genetic and Genomic Services in the Philippines

### Programs

The integration of NBS in the public health system has been an opportunity for introduction of genetic services in the 17 regions of the country. Currently, there are regional DOH NBS coordinators assisted by a full‐time NBS nurse coordinators who are also involved in other genetics‐related activities, such as the Birth Defects Surveillance (BDS) and the Telegenetics Referral system, which aims to further expand the delivery of genetic services in the country (Padilla et al. 2011a).

### Human resources and training in medical genetics

Nationally there are only ten trained geneticists, eight are in Metro Manila and two are in the provinces (one in Davao City and one in Cebu City) making a medical geneticist to population density ratio of about 1:11,751,625. To date, all clinical geneticists are pediatricians. Medical genetics has been a recognized specialty since 2000. There is no separate department of Medical Genetics. Only the Department of Pediatrics at the PGH has a separate section for Clinical Genetics in the whole country. The PGH Department of Pediatrics offers a 2‐year fellowship program in Clinical Genetics, designed to provide broad clinical exposure to areas of dysmorphology, biochemical genetics, cytogenetics, molecular genetics, and neonatal screening programs (Padilla 2008). Since 2011, the fellowship program requires its fellows to pursue an MS in Genetic Counseling.

The University of the Philippines, Manila, started the 2‐year Master of Science in Genetic Counseling (MSGC) program in 2011, as a response to the growing need for counseling services of two major DOH programs—the NBS program and the BDS program. It is open to nurses, doctors, and other allied health professionals. Their training allows them to (1) apply the basics of human genetics and the principles of medical genetics and genetic counseling to given clients; (2) provide supportive genetic counseling to families, serve as patient advocates, and refer individuals and families to community and/or local government support services; (3) serve as educators and resource for other health care professionals and for the general public; and (4) plan, develop, and evaluate genetic services programs (Laurino et al. 2011).

## Research Priorities in Medical Genetics and Genomics

A recent development in the country was the establishment of the PGC under the University of the Philippines System in 2009 (www.philippinegenomecenter.org). The PGC is a multidisciplinary institution that combines basic and applied genomics research for development of health diagnostics, therapeutics, preventive products, and improved crop, aquaculture, and animal varieties. The PGC collaborates and establishes linkages within and outside the country. The Center also implements and promotes research program‐driven agenda on identified priority areas of national need and of competitive advantage in order to achieve a leading position in the country, in the region, and the world. Research grants are made available for specific programs on health, biodiversity, agriculture, ethnicity, and forensics, and ethical, legal, and social aspects.

The health program's main objective is to apply genomics for the promotion of Filipino health and the prevention and management of public health problems. Research projects of the PGC Health Program focus on development of diagnostic kits for early diagnosis of infectious diseases (dengue, tuberculosis, chikungunya, lepstospirosis, malaria, schistosomiasis, and salmonella) and noncommunicable diseases (diabetes, cardiovascular diseases, colorectal cancer); genome web library for H1N1 for early diagnosis of disease outbreaks.

Several study groups have been created for specific diseases with genetics and genomics as the focus of the research collaborations. Collaborations among geneticists, endocrinologists, cardiologists, hematologists, neurologists, and otorhinolaryngologists from different institutions and centers locally and internationally are ongoing.

Clinicians and scientists involved in an X‐linked Dystonia Parkinsonism (XDP) collaborative study group are studying an X‐linked recessive disorder of combined dystonia–parkinsonism first reported in 1975 on patients from the island of Panay, Philippines (Lee Lillian et al. 2002). All cases described are of Filipino ethnic background, with maternal ancestry in this part of the country which suggests a single genetic founder and genetic homogeneity (Domingo et al. 2015). There are five disease‐specific changes (DSC1, DSC2, DSC3, DSC10, and DSC12), a 48‐bp deletion, and a SVA retrotransposon insertion found within “deep intronic” or intergenic DNA segments, or in a nonconventional exon of the TAF1 gene on Xq13.1 which have been identified in affected individuals (Nolte et al. 2003). However, studies have not revealed mutations in the coding regions of the gene and research is still focused on searching for the exact disease‐causing variant (Domingo et al. 2015).

Otitis media has also been a research focus in another international collaborative study because of its high prevalence among an indigenous Filipino community. Rare variants in alpha‐2‐macroglobulin‐like 1 (A2ML1) predisposing to nonsyndromic otitis media within different study populations have been described (Santos‐Cortez et al. 2015). The A2ML1 gene encodes a middle ear‐specific protease inhibitor, a marker for vascular permeability of middle ear mucosa during infection. In another related study on otitis media and the indigenous Filipino population, risk factors such as environmental variables, A2ML1 variant carrier status, and middle ear bacteria were analyzed. Findings have shown that the A2ML1 genotype is the strongest predictor of otitis media occurrence within this population (Santos‐Cortez et al. 2016).

## Patient Organizations and Public Education in Genetics

Geneticists partner with parent/patient organizations for genetic disorders. Geneticists provide lectures to parents and other health professionals for a better understanding of the genetic disorders and its impact to the patient, the family, and society in general.

The Down Syndrome Association of the Philippines, Inc. (http://dsapi.org/), a nonstock, nonprofit organization, was established in 1992 by a group of parents and physicians to offer support to families who have a child with Down syndrome. The organization offers parent counseling, seminars, lectures, free medical and dental clinic services, sports activities, and art activities. The Philippine Society of Orphan Disorders (www.psod.org.ph), a nonstock, nonprofit organization, was founded in June 2006 to serve as a central network for the advocacy and effective coordination of all viable efforts to sustain a better quality of life for the individuals with orphan or rare disorders in the Philippines.

A volunteer youth group has been established in 2009 to promote awareness among the young people with regards to healthy lifestyle and healthy pregnancies. The VYLH‐Philippines (http://vylhphilippines.blogspot.com/) is a network of leaders from different youth organizations based in universities and communities in the country, and was organized with the aim of mobilizing the youth toward health work. Currently, the network is doing advocacy and promotional work in their respective schools and communities, focusing on (1) raising national awareness about the importance of folic acid supplementation in the prevention of neural tube defects; (2) newborn screening for the prevention of mental retardation and death; and (3) support for Republic Act 10747, Rare Diseases Act of the Philippines, legislation that provides a comprehensive package of affordable and accessible health care for patients with rare diseases. (Padilla et al. 2012).

## Legislation

The IHG‐NIH has been instrumental in the preparation, lobbying, and implementation of two laws—the Newborn Screening Act of 2004, Republic Act No. 9228 (www.newbornscreening.ph/images/stories/ResourcesDOHPolicies/RA9288.pdf), and the Rare Disease Act of 2016, Republic Act 10747 (http://www.gov.ph/2016/03/03/republic-act-no-10747).

## Conclusion

The Philippines still faces many challenges in the delivery of services and the conduct of genetic and genomic research. Although one of the active countries in Southeast Asia with regard to genetics, the country still has a shortage of geneticists and genetic counselors. Difficulties exist for continued research and integration of health care services into the public health system due to limited resources. Geographical, cultural, and financial barriers must be overcome by the country in order to realize this goal. Despite these shortcomings, there is a promising future for medical genetics in the country with the help of the government and support of the community.

## Conflict of Interest

None declared.
